# Diabetes Mellitus

**Published:** 1842-07

**Authors:** 


*Diabetes Mellitus.*—M. Bouchardat read a paper on the treatment of this disease before the Academy of Sciences of Paris, Nov. 15, 1841. The most difficult point in the treatment of this disease is, Mr. B. states, to induce patients to abstain for a length of time, and completely, from all aliments containing fæcula. Although the patient may be firmly convinced that the use of bread is fatal to him, yet, in spite of remonstrance and the utmost vigilance of his attendants, he resumes the nutriment; the diabetic symptoms, which had been moderated during the abstinence from bread, now appear; tubercles are deposited in the lungs, and the patient dies.

In the treatment of diabetes mellitus two important points present themselves for the consideration of the physician; 1. To replace bread by a nutriment containing less fæcula; and 2. To restore the economy to its normal state.

The first of these indications M. Bouchardt has endeavored to fulfil by preparing bread with gluten. M. E. Martin had succeeded in separating the gluten during the manufacture of starch, but it was necessary to add one-fifth of flour to the gluten in order to make eatable bread. The bread thus prepared is light, and of an agreeable taste, and by using it with animal food the patient will take in little more than a scruple and a half of fæcula during the day.

With regard to the second indication the following considerations guided the author. In diabetes the acid secretion of the skin is suddenly and completely suspended; the mucous follicles and glands of the alimentary canal secrete fluids which are considerably altered in consequence of the change in the cutaneous transpiration, instead of being alkaline they are acid. But are we to conclude that this acid secretion has any influence on the conversion of fæcula into sugar? Certainly not; for the author had long ago ascertained that acids are incapable of producing any such effect at the temperature at which digestion takes place. Connected with this point, however, there is a fact of much importance. Whenever the organic acids, just alluded to, exist in any great quantity, we have joined to them that modification of albumen which assists in the transformation of fæcula into sugar. The same coincidence probably exists in the system of diabetic patients, and the chief circumstances to which we have to attend are the suppressed perspiration, and the perverted secretion of the digestive canal.

M. Bouchardat next alludes to the various attempts which have been made for the purpose of restoring the cutaneous perspiration; he has found no benefit from the vapor-baths, so highly praised by Dr. Bardsley and others, nor from the sulphureous baths, or the hydrosulphate of ammonia, recommended by Rollo. The best means of restoring the functions of the skin, according to the author, are—1. The use of flannel clothing, in sufficient quantity to keep up constant diaphoresis. 2. The internal use of sudorifics.—*Am. Jour. Med. Sci.*, from *Provin. Med. and Surg. Journ.*, Dec. 4, 1841.

